# Whole genomic comparative analysis of *Streptococcus pneumoniae* serotype 1 isolates causing invasive and non-invasive infections among children under 5 years in Casablanca, Morocco

**DOI:** 10.1186/s12864-020-07316-0

**Published:** 2021-01-07

**Authors:** Néhémie Nzoyikorera, Idrissa Diawara, Pablo Fresia, Fakhreddine Maaloum, Khalid Katfy, Kaotar Nayme, Mossaab Maaloum, Jennifer Cornick, Chrispin Chaguza, Mohammed Timinouni, Houria Belabess, Khalid Zerouali, Naima Elmdaghri

**Affiliations:** 1 Department of Microbiology, Faculty of Medicine and Pharmacy of Casablanca, Hassan II University of Casablanca, Casablanca, Morocco; 2 Bacteriology-Virology and Hospital Hygiene Laboratory, Ibn Rochd University Hospital Centre, Casablanca, Morocco; 3 Faculty of Sciences and Health Techniques, Mohammed VI University of Health Sciences (UM6SS) of Casablanca, Casablanca, Morocco; 4 Institut Pasteur de Montevideo, Pasteur + INIA Joint Unit (UMPI), Montevideo, Uruguay; 5 Institut Pasteur de Montevideo, Microbial Genomics Laboratory, Montevideo, Uruguay; 6 Molecular Bacteriology Laboratory, Institut Pasteur du Maroc, Casablanca, Morocco; 7 Laboratory of Biology and Health, Faculty of Sciences Ben M’Sik, Hassan II University of Casablanca, Casablanca, Morocco; 8 Aix Marseille University, IRD, AP-HM, SSA, VITROME, Marseille, France; 9 Malawi Liverpool Wellcome Trust Clinical Research Programme, Blantyre, Malawi; 10 Institute of Infection and Global Health, University of Liverpool, Liverpool, UK; 11 Wellcome Trust Sanger Institute, Cambridge, UK

**Keywords:** *Streptococcus pneumoniae*, Serotype 1, Whole genome sequencing, Comparative genomic analysis

## Abstract

**Background:**

*Streptococcus pneumoniae* serotype 1 remains a leading cause of invasive pneumococcal diseases, even in countries with PCV-10/PCV-13 vaccine implementation. The main objective of this study, which is part of the Pneumococcal African Genome project (PAGe), was to determine the phylogenetic relationships of serotype 1 isolates recovered from children patients in Casablanca (Morocco), compared to these from other African countries; and to investigate the contribution of accessory genes and recombination events to the genetic diversity of this serotype.

**Results:**

The genome average size of the six-pneumococcus serotype 1 from Casablanca was 2,227,119 bp, and the average content of coding sequences was 2113, ranging from 2041 to 2161. Pangenome analysis of the 80 genomes used in this study revealed 1685 core genes and 1805 accessory genes. The phylogenetic tree based on core genes and the hierarchical bayesian clustering analysis revealed five sublineages with a phylogeographic structure by country. The Moroccan strains cluster in two different lineages, the five invasive strains clusters altogether in a divergent clade distantly related to the non-invasive strain, that cluster with all the serotype 1 genomes from Africa.

**Conclusions:**

The whole genome sequencing provides increased resolution analysis of the highly virulent serotype 1 in Casablanca, Morocco. Our results are concordant with previous works, showing that the phylogeography of *S. pneumoniae* serotype 1 is structured by country, and despite the small size (six isolates) of the Moroccan sample, our analysis shows the genetic cohesion of the Moroccan invasive isolates.

## Background

*Streptococcus pneumoniae*, an encapsulated commensal bacterium of the human nasopharynx, remains a major cause of mortality and morbidity worldwide, that frequently infects children under 2 years old with immature immune system and elderly with a decrease of the immune response, as well as people with underlying diseases [1, 2]. This pathogen is the well-known agent of non-invasive infections such as sinusitis and otitis media, and invasive infections such as meningitis, bacteraemia and bacteraemic pneumonia [1]. According to the World Health Organization (WHO) in 2015, of the estimated 5.83 million globally deaths among < 5 years old children, 294,000 deaths were due to pneumococcal infections [2].

Nowadays, nearly 100 serotypes have been identified based on antibody binding to specific epitopes and on structural differences in its capsular polysaccharides [3]. Some serotypes are known to cause invasive diseases and others are mainly found in nasopharyngeal carriage [4]. Pneumococci expressing serotype 1 are often associated with invasive infections and are rarely found in carriage. This serotype can cause invasive pneumococcal disease (IPD) outbreaks in small or closed communities, and lethal meningitis epidemics [5, 6]. In African countries, serotype 1 remains a leading cause of IPDs, even in countries with pneumococcal conjugated vaccines (PCV), PCV-10/PCV-13 [5].

Pneumococcal vaccination constitutes the best option for preventing IPD, and in several countries, PCVs have been recommended in childhood immunization programs for more than a decade. They have significantly reduced the global IPD burden in children as reported in the review of Izurieta [7]. In Morocco, the national immunization program introduced PCV in October 2010 firstly by PCV-13 in 2 + 1 schedule at the age of 2, 4 and 12 months, then switched to PCV-10 in July 2012 in the same schedule. In Casablanca, the study conducted by Diawara and his colleagues on IPD among ≤5 years children showed the persistence of serotype 1 after introduction of PCVs [8].

Multi Locus Sequence Typing (MLST) is a current method used to characterize pneumococcal populations. Compared to this technique, whole genome sequencing (WGS) has a highest discriminatory power for bacterial genomes analysis. Indeed, WGS has increased power to establish the evolutionary relationships between close strains across the species [9]. Multiple studies have used the high-throughput WGS to investigate PCV impact and clustering pneumococcal populations into groups using genomic variation that reflect a recent evolutionary history [10–13]. The objectives of this study, as part of the Pneumococcal African Genome project (PAGe), were to determine the phylogenetic relationships of serotype 1 isolates recovered from children patients in Casablanca, compared to isolates from other African countries (Egypt, Ethiopia, Ghana, Malawi, Mozambique, Niger, Nigeria, South Africa and The Gambia), and to investigate the contribution of accessory genes and recombination events to the genetic diversity of this serotype.

## Results

### Characteristics of pneumococcal serotype 1 isolates from Casablanca, Morocco

We analyzed six strains of *S. pneumoniae* serotype 1 isolated among ≤5 years children (Table 1). Five isolates were from invasive infections while the remaining strain was isolated from the protected distal bronchial sample (PDBS) in the pneumonia context. Of these invasive strains, four were isolated from blood and the other from the cerebrospinal fluid (CSF). All isolates were susceptible to all antibiotics tested. The minimum inhibitory concentration (MIC) to penicillin G and ceftriaxone varied between [0.016–0.032] μg/ml for both beta lactams antibiotics.

**Table 1:** Sources and genome organization of *S. pneumoniae* serotype 1 isolates from Casablanca

Strain ID	Isolation year	Patient age (mounths)	Gender	Source	Genome length (bp)	CDS content
Morocco_1	2007	48	Female	Blood	2,210,078	2117
Morocco_2	2008	9	Female	Blood	2,237,618	2138
ni_Morocco_3	2009	60	Male	PDBS	2,319,720	2161
Morocco_4	2008	24	Male	Blood	2,218,009	2120
Morocco_5	2009	11	Male	Blood	2,199,811	2104
Morocco_6	2012	7	Male	CSF	2,177,478	2041

*PDBS* protected distal bronchial sample

*CSF* Cerebrospinal fluid

*CDS* Coding Sequence

### General genome features

The six strains of *S. pneumoniae* serotype 1 were sequenced using the Illumina HiSeq 2500 system (Table 1). The average length of the six pneumococcus serotype 1 genomes analyzed was 2,227,119 bp. The minimum genome length was 2,177,478 bp corresponding to the strain isolated from CSF, while the maximum genome size was of 2,319,720 bp for the strain isolated from PDBS. The CDS content ranged from 2041 to 2161 with an average of 2113. The minimum and maximum CDS content were of the strain from CSF and PDBS respectively.

### Phylogenomic analysis and population structure

**Fig. 1:**
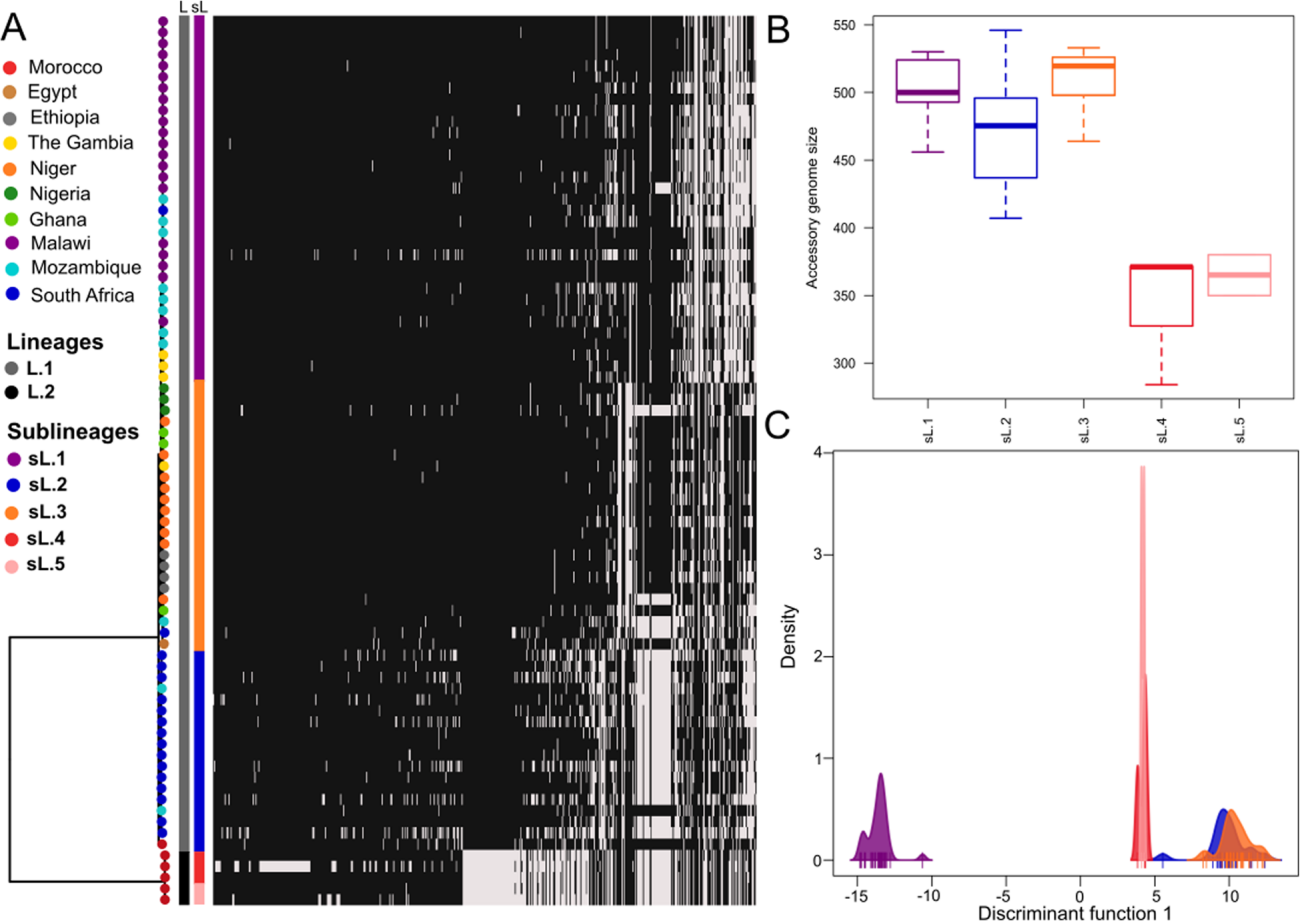
**a** Phylogeny of serotype 1 *S. pneumoniae* isolates. The tips of the tree are colored by country, and beside there are two bars that are the result of the clustering analysis with hierBaps. One bar, grey and black, represent the Lineages; and the other, with five colors, represent sublineages. The heatmap are the accessory genes, with no singletons. **b** The boxplots shows the accessory genomes size, with singletons. **c** shows a DAPC analysis

The maximum likelihood reconstruction of the phylogenetic relationships among the African isolates of *S. pneumoniae* serotype 1 based on the core genome, and the hierarchical bayesian clustering analysis revealed 2 lineages and five sublineages. The five sublineages population structure clusters the isolates by country and geographic region (Fig. 1a). With regard to the Moroccan strains, the phylogenetic analysis showed a basal divergent clade with the five invasive strains that clusters in two sublineages, and is distantly related to the non-invasive strain (ni_Morocco_3) that is highly related with the other African genomes (Fig. 1a). All the five invasive moroccan strains were of ST306 while the remaining non-invasive strain was of ST2084. The identified ST306 matched the lineage 2 exclusively associated with invasive strains.

### Pangenome analysis

**Fig. 2:**
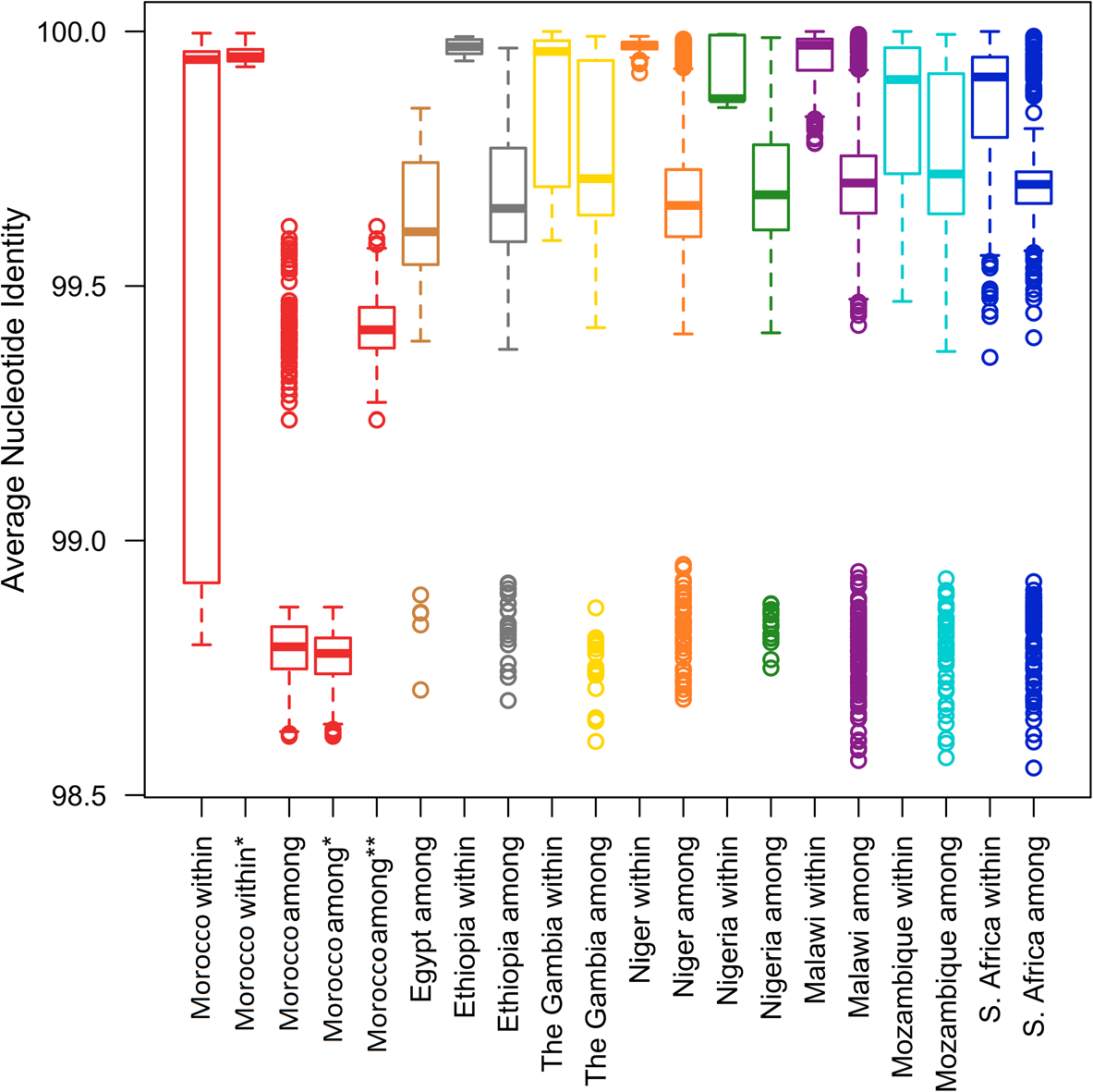
ANI values obtained comparing the *S. pneumoniae* genomes. The colors are by country following Fig. 1. Morocco within = all Moroccan genomes, Morocco within* = all Moroccan genomes without ni_Morocco_3, Morocco among = all Moroccan genomes against all other genomes, Morocco among* = all Moroccan genomes without ni_Morocco_3 against all other genomes, Morocco among* = ni_Morocco_3 against all other genomes from other countries

To explore the accessory genes composition of *S. pneumoniae* serotype 1, we reconstructed the pangenome of the 80 genomes. The analysis revealed a total of 3490 genes, from which 1685 were core genes, and 1805 accessory genes from which 1311 and 494 represented shell and cloud genes respectively (Table 2). Figure 1a shows the differences in the accessory genes distribution across sublineages, and Fig. 1b explicit that the accessory genomes sizes in invasive sublieanges are substantially smaller. Finally, we can see in Fig. 1c based on the discriminant analysis of principal components (DAPC) using the accessory genes that sublineage 1 is highly different from the remaining sublineages. Sublineages 2 and 3, as sublineages 4 and 5, are overlapped in the accessory genes composition (Fig. 1c). Despite the high divergence observed among the strains in the accessory genes composition, they are still being the same species. All ANI (Average Nucleotide Identity) values are all > 95%, which value is indicative of the same species of pneumococcus (Fig. 2).

**Table 2:** Pangenome analysis of 80 genomes of *S. pneumoniae* serotype 1 strains

Gene types	Average covering	Number of genes
Core genes	95% < = strains <= 100%	1685
Shell genes	15% < = strains < 95%	494
Cloud genes	0% < = strains < 15%	1311
Total genes	0% < = strains <= 100%	3490

### Recombination detection

**Fig. 3:**
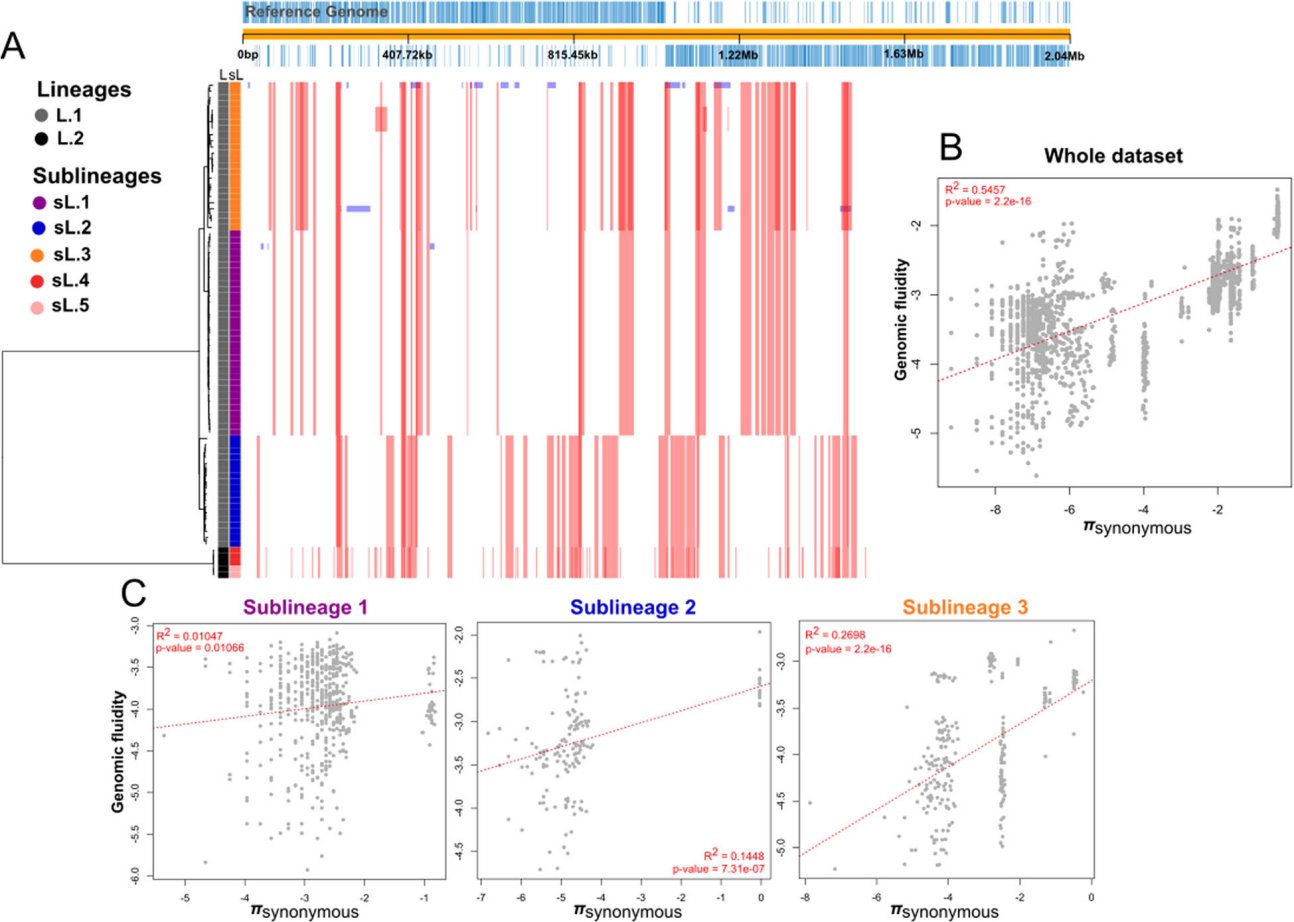
Recombination and accessory genome analysis of *S. pneumoniae* serotype 1 isolates. **a** Recombination using Gubbins: The tree was built from non-recombinant regions in the core genome alignment. The colored strip highlights the BAPS clusters. Red blocks indicate recombinant regions along the core genome. Blue blocks indicate recombination event that have occurred in a single isolate. **b** Linear regression analysis of genomic fluidity (accessory genome) against synonym diversity (core genome). Plotting the values are in ln-ln. **c** Same as **b** but done by sublineage; Sublineage 1:*R*^2^ = 0.01047, *p*-value = 0.01066, Sublineage 2: *R*^2^ = 0.1448, *p*-value = 7.31e^− 7^, Sublineage 3: *R*^2^ = 0.2698, *p*-value = 2.2 e^− 17^. Sublineage 4 and 5 were not plotted because they have a limited value due to the small number of isolates

The recombination events on the core genome alignment of the 80 isolates were examined to investigate their contributions to the phylogenetic diversity of serotype 1. Our results shows that all African *S. pneumoniae* serotype 1 lineages underwent multiple recombinations across its evolutionary history (Fig. 3a). The two sublineages (i.e. sL.4 and sL.5) identified in the invasive Moroccan isolates underwent many recombination events among it, but also with isolates of sL.2 where the non-invasive Moroccan isolate clusters. We also correlated the Jaccard distance, as a measure of the dynamic and similarty of the accessory genome, with the nucleotide diversity based on the synonymous polymorphisms (neutral evolution). As shown in Fig. 3b for whole dataset, R2 = ~ 0.5 and is significative, at hence, there is no signal of adaptive evolution. But looking at sublineages level (Fig. 3c), sL.1, sL.2 and sL.3 did not show correlation among Jaccard distance and the core genome synonymous diversity (πsynonymous) indicating adaptive evolution of accessory genome. The results are not conclusive for the Moroccan isolates due to the few isolates, sL.4 (three isolates) and sL.5 (two isolates).

## Discussion

Recent advances in genome sequencing technologies and the emergence of WGS use in genome comparative studies have provided powerful tools to study the genomic diversity of bacterial pathogens by using genome sequence data. In this study, we made the genomic comparison of six clinical *S. pneumoniae* serotype 1 isolates from Morocco to those from other African countries by using WGS. Serotype 1 still the main cause of IPD in developing countries. In African countries, serotype 1 is one of the top three of most common vaccine serotypes causing IPD among children [14, 15].

All Moroccan isolates were susceptible to all antibiotics tested. Serotype 1 is rarely linked with antibiotic resistance. This could be associated with the short duration of this serotype in asymptomatic colonization of the human nasopharynx, where exchange of genetic elements through recombination with other Streptococci took place [16–18].

The average length of the genome of the serotype 1 strains from Morocco was 2,227,119 bp. This genome size is greater than the size of the reference strain of serotype 1, *S. pneumoniae* P1041 (accession number: FQ312030) which is 2,142,122 bp. In addition to that, the average genes content found in Moroccan strains was 2113 in the range of 2041 to 2161 CDS. Compared to CDS content of *S. pneumoniae* P1041 (1824 CDS), the gene content of Moroccan isolates was higher. The difference of Moroccan strains in genome length and CDS content could be explained by the frequent occurrence of horizontal gene transfer events among the genus *Streptococcus* [19].

In our study, 1685 CDS were highlighted as core genome in all 80 isolates analyzed which corresponds on 79.7% of the total genome translation. The quantification of the bacterial core genome may vary among different isolates collections. Moreover, it is highly dependent on the core genome definition as well as the parameters fixed for the pangenome analysis [20]. In many cases, the core genome is made of genes involved in essential functions such as protein synthesis, DNA related metabolism and cellular processes [20]. The accessory genome of the serotype 1 isolates studied was highly variable. The pangenome analysis by Roary revealed a total of 1805 accessory genes found in 0% < = strains < 99% of the 80 strains analyzed. Those genes are mainly associated with evasion of the host immune system and virulence factors [21]. Nevertheless, our findings showed that the accessory genomes in invasive sublineages were substantially smaller. This result could be associated with low recombination rate in invasive strains as the human nasopharynx is the main reservoir of genetic exchanges driven by recombination, horizontal gene transfer and events of gene loss or addition occur. The IPD associated genes have been reported shorter than those in the soft-accessory genome [22].

Phylogenetic tree and the hierarchical bayesian clustering analysis revealed 2 lineages and five sublineages with a geographic structure by country. In the large study conducted on the whole genome phylogeny of serotype 1 pneumococcal isolates [23], the authors showed a high level of geographical structure especially among African isolates, with multiple inter country transfers between bordering countries, in particular Mozambique, Malawi and South Africa. Many phylogenetic studies worldwide grouped different pneumococcal collections in many clusters [18, 24, 25]. The study on phylogenetic analysis in South Africa by du Plessis et al.(2016) [26], the global invasive serotype 1 population was grouped in 9 clusters and showed a strong phylogeographic structure. According to our phylogenetic tree, the invasive strains from Morocco formed one clade, suggesting that there are genetically cohesive. Moreover, some genes were absent only in that clade. Since pneumococcal strains have substantial genomic variation, the locations of genes within their respective genomes are not constant [27].

The sublineage 1 is exclusively formed by strains from Malawi and Mozambique. This sublineage is highly different from the remaining sublineages based on the DAPC using the accessory genes. As reported elsewhere, the Malawi and Mozambique serotype 1 pneumococci formed a single and genetically stable clade over the sampling period without detectable impact from imported clones [23]. As published by Chaguza et al. [28], the identified sequence Clusters (SCs) in African pneumococcal serotype 1 population matched the phylogenetic clades from the phylogeny and were predominantly associated with geographical origin of the isolates. With regards to Moroccan isolates, the identified ST306 matched the lineage 2 exclusively associated with invasive strains. The ST306 is a worldwide distributed pneumococcal serotype 1, suspected to be an important pathogen behind the increase of the serotype 1 IPD expansion and is responsible for more than 80% of cases of serotype 1diseases [29, 30].

As expected, the strains analyzed underwent multiple recombinations. *S. pneumoniae* is naturally transformable and recombination events play a major role in its molecular evolution [31, 32]. Some studies reported low presence of recombination in serotype 1, and that the rates with which this phenomenon occurs vary greatly among pneumococcal serotypes and lineages [33, 34]. Compared to invasive strains, the strain isolated from PDBS were associated with the largest genome and the highest number of CDS as well as the accessory genes. This finding could suggest that the strain was probably carried for long periods enough to result in extensive genetic exchanges with other closely related species as *Streptococcus mitis* as reported in the large study of Lessa et al. [17]. In that study, *wzy1* operons of *S. mitis* clones were found highly similar to *wzy1* of the serotype 1 *S. pneumoniae* reference strain.

## Conclusions

This study constitutes the first pneumococcal genome analysis by whole genome sequencing in Morocco, providing increased resolution on the analysis of the highly virulent serotype 1. Our results confirmed the phylogeographic structure by country of *S. pneumoniae* serotype 1 despite the small size of the Moroccan sample, and showed it basal position on the phylogenetic tree of African isolates. The Moroccan isolates are structured in three sublineages, with the non-invasive strain which clusters in sL.2 being highly divergent from the invasive strains (sL.4 and sL.5).

## Methods

### Bacteria isolates

The microbiology laboratory of Ibn Rochd University Hospital Centre (IR-UHC) of Casablanca carries out surveillance of invasive and non-invasive pneumococcal infections in children ≤5 years [8]. All pneumococcal strains were isolated and identified according to the standard bacteriology procedures. Serogrouping was done by the checkerboard method with Pneumotest-latex (Statens Serum Institute antisera, Copenhague, Denmark). Serotyping was performed by Quellung capsule swelling using Statens Serum Institute antisera (Statens Serum Institute antisera, Copenhague, Denmark).

Antibiotic susceptibility tests were performed on Mueller-Hinton agar additioned with 5% of sheep blood (BioMérieux, Marcy-l’Etoile, France) and interpreted according to the Clinical Laboratory Standard Institute (CLSI, 2012) recommendations [35]. Oxacillin (1 μg) was used for screening of penicillin non-susceptible *S. pneumoniae*. Erythromycin, chloramphenicol, clindamycin, vancomycin, cotrimoxazole, rifampicin, tetracycline and levofloxacin were tested by disc diffusion method. The MIC of penicillin G and ceftriaxone were determined by E-test method with E-Tests from Oxoid (Oxoid, Basingstoke, UK) on Mueller-Hinton agar additioned with 5% of sheep blood (BioMérieux, Marcy-l’Etoile, France). The breakpoints recommended by CLSI in 2012 were used for interpretation: ≤ 0.06 μg/ml and ≥ 2 μg/ml for penicillin, ≤ 0.5 μg/ml and ≥ 2 μg/ml, for ceftriaxone for meningeal isolates and ≤ 1 μg/ml and ≥ 4 μg/ml for non-meningeal isolates. Quality control was conducted using *S. pneumoniae* ATCC 49619.

From 2007 to 2014, 9 strains (invasive and non-invasive) of serotype 1 were isolated in children under 5 years old. Three of them were lost. Six (6) isolates (5 invasive and 1 non-invasive) of *S. pneumoniae* serotype 1 causing infections among children under 5 years, were randomly selected from the data bank of the microbiology laboratory of IR-UHC of Casablanca, to perform the WGS analysis. All isolated strains were stored in brain heart infusion broth with 15% of glycerol at − 80 °C.

### Bacterial DNA preparation and whole genome sequencing

The genomic DNA of the six strains selected for this surveillance was purified with the QIAamp DNA Mini Kit (Hilden, Germany) following the manufacturer’s recommendations. DNA quality and quantity were estimated by measuring the absorbance of the sample using NanoVue™ Plus Spectrophotometer (GE Healthcare UK Limited, UK) at wavelengths 260 nm and 280 nm following the manufacturer’s instructions. Extracted DNA were stored at − 20 °C. The DNAs of the six strains were whole-genome-sequenced using an Illumina HiSeq 2500 platform at the Wellcome Trust Sanger Institute, as part of the PAGe project. Libraries were constructed using the Nextera XT DNA Library Preparation Kit and sequenced with the HiSeq Reagent Kit (pair-end reads of 150 bp).

### Genome assembly and annotation

The quality of the generated reads from high throughput NGS was assessed using FastQC v0.11.8 [15]. After removal adaptor sequences, reads of each isolate were de novo assembled using SPAdes v3.11.1 [36] with a k-mer size automatically determined by the package. The obtained draft assemblies were annotated using the Prokka (Prokaryotic annotation) software, which predicts genes, based on available annotation informations such as proteins and coding sequences (CDS) [37]. Average Nucleotide Identity (ANI), a whole-genome similarity metric was used to investigate the relatedness among isolates genomes.

### Recombination, phylogenetic and population structure analysis

In the study of by Chaguza et al. [28], the phylogenic analysis of the global population structure of serotype 1 in Africa showed that all isolates were grouped in five distinct clades. From those clades, we selected a balanced genomic data of 74 public genomes of serotype 1 from nine African countries (Egypt, Ethiopia, Ghana, Malawi, Mozambique, Niger, Nigeria, South Africa and Gambia) previously published [28]. Data were extracted in the European Nucleotide Archive (ENA) database (Additional file 1). Recombination was analyzed among the strains from Morocco, and 74 public genomes of serotype 1 using Gubbins algorithm [38] over the core genome alignment generated by progressiveMauve [39], a software package that attempts to align orthologous and xenologous regions among genome sequences. First, we removed inconsistent alignment columns with trimAl [40] in all concatenated locally collinear blocks, and then Gubbins was run over the core genome alignment. For the inference of the phylogenetic relationships among the 80 isolates, Maximum Likelihood (ML) phylogenetic analyses were performed by using RAxML v8.2.12 [41] based on core genome obtained with progressiveMauve (recombinations were filtered out with Gubbins), with 1000 bootstrap iterations. The clustering analysis was done with hierBaps (Bayesian clustering tool for population genetics). Sequence types (STs) of moroccan *S. pneumoniae* isolates were determined by the sequences of seven housekeeping genes (*aroE, gdh, gki, recP, spi, xpt, and ddl*) obtained from the results of WGS. Allelic numbers and STs were assigned by using the pneumococcal Multilocus Sequence Typing (MLST) website (https://pubmlst.org/spneumoniae/).

### Pangenome reconstruction

To accurately reconstruct the pangenome of the whole dataset, all 80 assembled and annotated genomes (6 genomes from Morocco and 74 public genomes) were analyzed by Roary v3.11.2 [42]. DAPC [43] was used to investigate the accessory genes distribution among sublineages in order to explore its differences.

Finally, we did a linear regression of the Jaccard distance based on the accessory genes and the nucleotide diversity of synonym sites of core genes for each pair of genomes to provide insights of accessory genome adataptive evolution. The analysis was done using the R package pagoo (https://github.com/iferres/pagoo), computing the Jaccard distance between each pair of organisms by the vegan::vegdist function [44] and the pairwise nucleotide diversity by the pegas::nuc.div function [45].

## Supplementary Information

**Additional file 1.**

## Data Availability

Sequence information of the 6 Moroccan pneumococcal serotype 1 genomes are available in the European Nucleotide Archive (ENA) database under the accession number given in Additional file 1. The 74 public genomes of *S. pneumoniae* serotype 1 used for the phylogeny reconstruction in this study were all downloaded from the ENA database; their accession numbers are listed in Additional file 1.

## Abbreviations

CDS: Coding DNA sequence
BAPS: Bayesian clustering tool for population genetics
DAPS: Discriminant analysis of principal components
ANI: Average Nucleotide Identity
ENA: European Nucleotide Archive
CLSI: Clinical Laboratory Standard Institute
WHO: World Health Organization
PCV: Pneumococcal conjugated vaccines
IPD: Invasive pneumococcal diseases
WGS: Whole genome sequencing
PDBS: Protected distal bronchial sample
CSF: CEREBROSPINAL fluid
PFGE: Pulse Field Gel Electrophoresis
MLST: Multi Locus Sequence Typing
ST: Sequence Type
MIC: Minimum inhibitory concentration
SNPs: Single nucleotide polymorphisms
